# Why We Still Need HBV Population-Based Epidemiologic Studies

**DOI:** 10.5812/hepatmon.842

**Published:** 2012-02-29

**Authors:** Payman Adibi

**Affiliations:** 1 Department of Gastroenterology and Liver Diseases, Isfahan University of Medical Sciences, Isfahan, IR Iran

**Keywords:** Epidemiology, Pupulation, Hepatitis B virus

As a practicing physician, I face clinical problems every day. To solve them, I use clinical decision making skills including reaching the most accurate diagnosis and deciding on the next step or steps to manage the problem. A clinician is a pragmatic person; i.e., s/he needs practical, implementable data to guide reasoning or decision making and nothing else! In this environment, why is Mohammad Reza Ghadir's article on the epidemiology of Hepatitis B virus infection in this issue of the journal at all? [1]. Of course it is a Socratic question, and you may find me to be the author of similar articles [2]. Description and research on epidemiology from the aspects of incidence and prevalence will affect pre-test likelihood in clinical decision-making. When a doctor encounters a patient in daily practice, s/he needs to know the possibility of the presence of a condition or disease when a sign or symptom is present; therefore the background epidemiology of the disease in the population that the patient comes from is important. It is especially important when there are limited data regarding the population; for example when there is a demographic shift due to immigration to a new area or a change of transmission-related factors after universal HBV vaccination. In terms of infectious disease, these epidemiologic shifts may be rapid and may necessitate surveillance and trend analysis through point estimation studies. In the context Ghadir reported, there is a dynamic, ever-migrating population and a disease whose epidemiology changes frequently. Epidemiologic studies may help indicate possible risk factors, especially when using methodologies that include attributable risk calculations. The risk factors may be manageable in a clinical setting or may be used by health authorities to plan preventive projects for the community. This approach may be combined with an estimation of risk factor manipulation in the patient as a single case as well as in the community. For example, if there is an established attributable risk of intravenous drug use with needle sharing that could disseminate the infection in the community, you may put an HBV patient on Methadone maintenance therapy (MMT). On the other hand, in a case of monogamous sexual contact with an HBcAb-positive sex partner at the age of 50, you may conclude that there is no need to recommend barrier methods. Along these lines, the risk factor tables in the Ghadir articles attempt to guide us. This is a methodological change and results in a nested case-control type of plan within a large cross-sectional study. When the prevalence of a disease is low and consequently the cases within a sample of population are limited, analyzing risk factors will result in methodological problems. This is the problem that Ghadir encounters in some rows of his table, for example when analyzing tattooing as a risk factor. On other hand, when there is a shared risk factor in the majority of the sample and there is no measurement of higher or lower exposure rates, the researcher cannot conclude that this factor is essentially a risk or not. Consider unsafe barber practices within a male population and its effect on HBV transmission. The last evidence-gathering step before concluding on clinical guidelines would be a decision analysis study. The formal methodology in such studies is to draw a decision tree and conduct reverse calculations. To enable the calculation, probabilities are needed for each arm, and to define the probabilities, it is essential to know the prevalence and incidence of each subgroup, which are extractable from epidemiologic studies. Even when a researcher intends to do a sensitivity analysis as part of the decision analysis, the ideal conclusion will be reached when the epidemiologic variations are known and a systematic review performed, such as that done by Alavian et al. in their publication on HBV epidemiology [3]. In terms of data consistency, a new piece of information on the same HBV epidemiology surely does not add anything to our current preventive and clinical practice; in such circumstances when associated with limited manpower and financial support, resources are better shifted toward other studies. Along with the importance of population based epidemiologic studies, I would like to note that, in terms of scientific reasoning, dissociating such studies from other biomedical studies on infectious transmission and risk factors may result in a reductionist philosophy. Without knowing the etiopathogenesis of the disease, variations in humans from the viewpoint of genomics, proteomics, and even metabolomics will result in pitfalls that have happened throughout the history of medicine.
